# Reappraisal of the glycerol test in patients with suspected Menière’s disease

**DOI:** 10.1186/1472-6815-14-12

**Published:** 2014-11-24

**Authors:** Bernd Lütkenhöner, Türker Basel

**Affiliations:** ENT Clinic, Münster University Hospital, Münster, Germany

## Abstract

**Background:**

Recent advances in magnetic resonance imaging make it possible to visualize the presumed pathophysiologic correlate of Menière’s disease: endolymphatic hydrops. As traditional diagnostic tests can provide only indirect evidence, they are hardly competitive in this respect and need to be rethought. This is done here for the glycerol test.

**Methods:**

The data of a previous retrospective analysis of the glycerol test in patients with suspected Menière’s disease are reinterpreted using a simple model. The mean threshold reduction (MTR) in the frequency range from 125 to 1500 Hz (calculated from audiograms obtained immediately before and four hours after the glycerol intake) is used as the test statistic. The proposed model explains the frequency distribution of the observed MTR by the convolution of a Gaussian probability density function (representing measurement errors) with a template representing the frequency distribution of the true MTR. The latter is defined in terms of two adjustable parameters. After fitting the model to the data, the performance of the test is evaluated using receiver operating characteristic (ROC) analysis.

**Results:**

The cumulative frequency distribution of the observed MTR can be explained almost perfectly by the model. According to the ROC analysis performed, the capability of the currently used audiometric procedure to detect a glycerol-induced threshold reduction corresponds to a diagnostic test of rather high accuracy (area under the ROC curve greater than 0.9). Simulations show that methodological improvements could further enhance the performance.

**Conclusions:**

Owing to their ability to reveal functional aspects without an obvious morphological correlate, traditional test for Menière’s disease could be decisive for defining the stage of the disease. A distinctive feature of the glycerol test is that it is capable of determining, with high accuracy, whether the pathophysiologic condition of the inner ear is partially reversible. Prospectively, this could help to estimate the chances of specific therapies.

## Background

In 1861, Prosper Menière reported on patients who suddenly suffered from intermittent attacks of vertigo combined with tinnitus and a gradually increasing hearing loss [1]. Although more than 150 years have passed since then, the disease, now named after him, is still not fully understood, and the criteria for establishing the diagnosis have not fundamentally changed. According to the widely accepted guidelines of the Committee on Hearing and Equilibrium of the American Academy of Otolaryngology - Head and Neck Surgery [2], the diagnosis of *definite* Menière’ disease requires (1) two or more definitive spontaneous episodes of vertigo 20 minutes or longer, (2) an audiometrically documented hearing loss on at least one occasion, (3) tinnitus or aural fullness in the treated ear, and (4) the exclusion of other causes; *probable* Menière’ disease is diagnosed if there is only one definite episode of vertigo. These definitions show that, as yet, the identification of Menière’s disease is largely dependent on the patient’s medical history. By implication this means that the numerous efforts to develop a specific diagnostic test [3, 4] did not lead to a practice that gained general acceptance. Recently, however, a major breakthrough was achieved. Using magnetic resonance imaging (MRI) with gadolinium as the contrast agent, Nakashima et al. [5] succeeded to visualize the presumed pathophysiologic correlate of Menière’s disease: endolymphatic hydrops. According to the above-mentioned guidelines, the diagnosis of *definite* Menière’s disease becomes *certain* by such confirmation, which hitherto could be obtained only after death. Meanwhile, this seminal work has been confirmed in many subsequent studies, in which the methodology was not only improved [6, 7], but also applied to specific questions [8–11].

In an MRI study by Fiorino et al. [12], each of 26 patients diagnosed with definite Menière’s disease showed evidence of endolymphatic hydrops exclusively in the affected ear. Moreover, there was no such evidence in 11 of 12 patients with other inner ear diseases. Considering the conclusiveness of these results, it can be expected that MRI will soon be the method of choice if a suspected diagnosis of Menière’s disease is to be confirmed by proving the hydrops. This intriguing progress appears to eliminate the need for other diagnostic procedures. However, such a conclusion would be premature. Diagnostic tests should be appraised in terms of their ability to improve patient-important outcomes [13], and in this respect, some of the traditional methods (or a combination of them) may ultimately turn out to be competitive, especially since it is not clear how important it is to prove endolymphatic hydrops in patients that were already diagnosed with definite Menière’s disease. If the above-mentioned results are representative, meaning that patients so diagnosed nearly always have endolymphatic hydrops (a supposition that would be consistent with Merchant et al. [14]), verifying the hydrops by whatever method provides hardly any new information. Thus, in future, more emphasis should probably be placed on the question as to what the various diagnostic tests can tell us about the stage and manifestation of the disease and to what extent they allow us to predict the prospects of specific therapeutic measures, e.g., treatment with betahistine [15].

As proving endolymphatic hydrops appears to become the domain of imaging techniques, the possible future roles of other diagnostic tests for Menière’s disease need to be rethought. This is done here for the glycerol test devised by Klockhoff and Lindblom [16], but some basic conclusions appear to be valid for other diagnostic procedures as well. The test exploits the fact that, in patients suffering from Menière’s disease, oral application of glycerol can temporarily improve the threshold of hearing, whereas no systematic effect is to be expected in patients with other hearing disorders and subjects with normal hearing. The underlying idea is that the dehydrating effect of glycerol transiently reduces the endolymphatic volume, which in turn may lead to partial recovery from hearing loss. To test for the latter, a pre-test audiogram is compared with an audiogram taken a few hours after the glycerol intake. While a significant threshold reduction can be regarded as evidence of endolymphatic hydrops, the reverse is not true: Since Menière’s disease is typically fluctuating and progressive [17, 18], there may be hydrops despite a negative glycerol test. It is known, for example, that the probability of a positive glycerol test depends on the phase of the disease, being minimal at times of remission [19]. Moreover, the hearing loss may be irreversible at a more advanced stage so that reducing the endolymphatic volume has no effect anymore.

Several variants of the glycerol test have been proposed since its first description, and so it seems timely to scrutinize the conceptual and methodological details of the test. In a previous article [20], we presented a retrospective study of 356 cases with suspected Menière’s disease (all ears fulfilled the aforementioned criteria for definite or at least probable Menière’s disease). In addition to descriptive analyses of the data, we introduced a new criterion for a positive test result. Moreover, we proposed a rule of thumb that can be used to define a subpopulation of patients for whom the probability of a positive outcome is significantly higher than for the excluded patients. The rule proved to be competitive with more advanced predictive modeling approaches [21]. However, gaining a deeper understanding of the test was impeded by the fact that there is no “gold standard” to compare with and that the determination of the auditory threshold is, like any measurement, affected by errors. In the present work, these problems are overcome by fitting a simple model to the data. The model gives an idea of what the results would be if the thresholds of hearing were determined exactly. Moreover, it becomes possible to assess the performance of the test by considering its receiver operating characteristic (ROC) curve and to predict what would be gained by methodological amendments.

## Methods

### Data

The same data as in our previous study [20], now available from a Digital Repository [22], are used. Briefly, archived audiograms from 347 patients that underwent a glycerol test to confirm a suspected Menière’s disease were transcribed into a computer-readable form. The tests had been performed following the protocol suggested by Klockhoff [19], which means that glycerol (1.2 ml/kg body weight) was orally administered with an equal amount of isotonic saline solution. The audiograms were obtained immediately before the glycerol intake (pre-test audiogram) and at hourly intervals thereafter (the last one obtained after four hours). Since *both* ears were investigated in a few patients, 356 cases are available altogether. But to restrict the data range to be plotted, two cases are excluded here as outliers (apart from that, the exclusion has no relevant impact on the results).

The effect of the administered glycerol is assessed by comparing the pre-test audiogram with the audiogram that was obtained after four hours. In the previous study [20], the aggregate threshold reduction (ATR) in a contiguous frequency range was used as a summary measure. But this quantity is inconvenient for modeling, because its calculation requires to integrate over a variable frequency range (the bounds of integration depend on the true hearing losses at the different frequencies as well as measurement errors), which makes it difficult (if not impossible) to apply standard statistical techniques. Therefore an alternative summary measure is used here: the mean threshold reduction (MTR) at the five lowest audiometric frequencies (125, 250, 500, 1000, and 1500 Hz), which represent the frequency range where the effect of glycerol is typically most pronounced. A convenient side-benefit of focusing on these frequencies is that the MTR is always an integer number (five thresholds are averaged, each of which was determined in steps of 5 dB).

**Figure 1:**
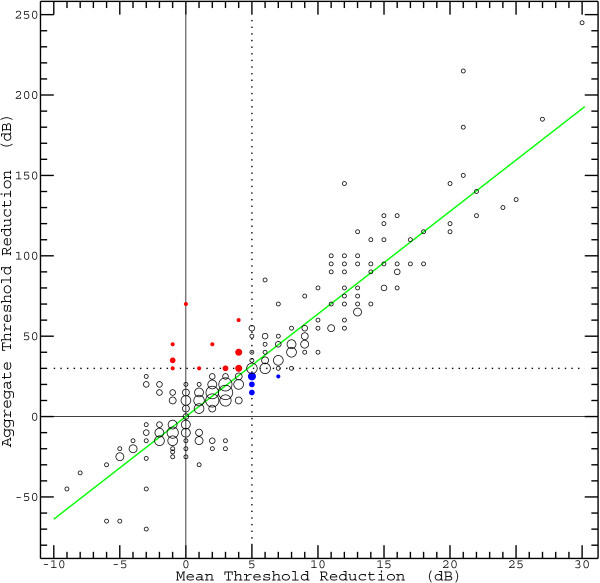
**Correlation between mean threshold reduction (MTR) and aggregate threshold reduction (ATR).** Both the MTR and the ATR can assume only a limited number of values. As a consequence, there are generally multiple occurrences for each combination of these measures so that a standard scatter plot would be problematic. The problem was solved by plotting a circle for each MTR-ATR combination and adjusting the radius so that the area of the circle is proportional to the number of occurrences. The two dotted lines (one horizontal, the other vertical) represent criteria that generally lead to consistent decisions as to the presence of a glycerol induced effect. The few exceptions are marked by filled circles: Blue indicates that the MTR is equal to or greater than the associated criterion value while the ATR falls short of the corresponding threshold. Red indicates that the situation is just the other way round.

Figure 1 shows that MTR (abscissa) and ATR (ordinate) are highly correlated (*R* = 0.924). In principle, each of the 354 cases considered in this study is represented by a single point, but the points partially coincide. Thus, instead of single points, circles with an area proportional to the number of points sharing the respective location are plotted. If the criterion for a positive glycerol test is that the ATR is at least 30 dB (dotted horizontal line), the false-positive rate may be expected to be about 5% [20]. Consistent decisions would be made by requiring the MTR to be at least 5 dB (dotted vertical line), apart from the few cases represented by the filled circles: In 16 cases (red circles) the test would be positive only according to the ATR-based criterion, and in 9 cases (blue circles) it would be positive only according to the MTR-based criterion.

### Convolution model

Audiograms measured at different times typically show discrepancies even when there is no reason to assume that the true threshold of hearing has changed. This intrinsic uncertainty of the threshold estimation may be considered as a measurement error, which, of course, propagates to every audiogram-based measure. As a consequence, the distribution of the observed MTR values reflects, to a considerable extent, the measurement error rather than the glycerol-induced threshold reduction. If the measurement error is assumed to be additive to the glycerol effect, the problem can be described by a convolution formula,
1

where *f*(*x*), *g*(*x*), and *h*(*x*) are probability density functions. The first one, *f*(*x*), characterizes the distribution of the MTR values actually observed, whereas the second one, *g*(*x*), characterizes the distribution that would be observed under ideal conditions, i.e., in the absence of measurement errors. The third function, finally, is the probability density function of the measurement error. In what follows, the measurement error will be assumed to be normally distributed, with a standard deviation estimated from the data. Given *h*(*x*), the unknown *g*(*x*) could be calculated by deconvolving the observed *f*(*x*), at least in theory. However, to be able to use this approach for the problem at hand, the number of cases would have to be increased by at least an order of magnitude [23, 24]. Thus, Eq. (1) will be used here in a different way. The idea is to “guess” a suitable function *g*(*x*) and to determine the parameters of this function so that the right-hand side of the equation optimally explains the observed *f*(*x*).

**Figure 2:**
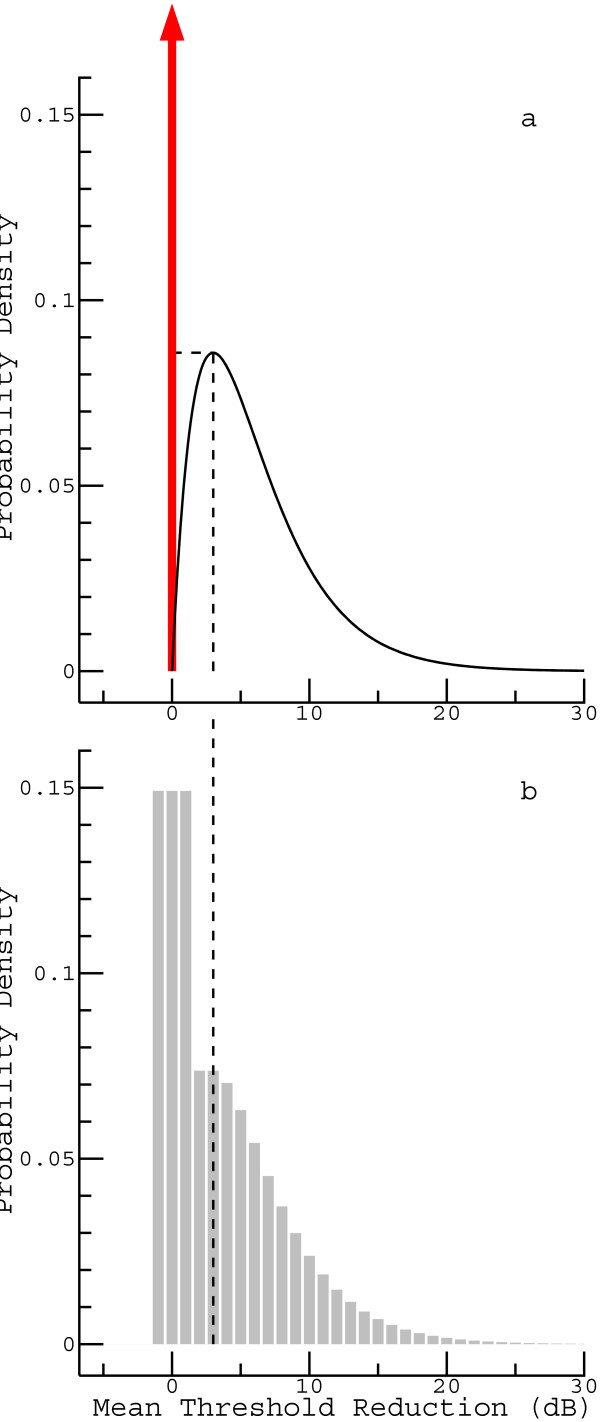
**Model for the distribution of the “true” MTR (i.e., the MTR that would be obtained if thresholds were estimated without errors). (a)** Basic idea. A first model parameter corresponds to the proportion of patients *without* a glycerol-induced threshold reduction (represented by the arrow), whereas a second one scales the MTR distribution of the patients showing an effect. **(b)** Discretized and smoothed version of the upper model.

As will be shown, the data can be explained reasonably well by means of an empirical function *g*(*x*) depending on only two parameters. The basic idea is outlined in Figure 2a. Conceptually, the patients are divided into two groups. Patients belonging to the first group, represented by the arrow in the figure, are assumed to show no glycerol-induced effect at all. Their proportion is denoted as *p*_0_ (in Figure 2 having a value of 0.3). Patients belonging to the second group are assumed to have a threshold reduction that is distributed according to a gamma distribution with a shape parameter of 2 (the choice of this well-known distribution was a pragmatic decision; other distributions with similar properties could be assumed as well). The corresponding probability density function is, for *x* ≥ 0,

2

where *θ* is called the scale parameter. Figure 2a shows this function for *θ* = 3. For reasons that will be explicated in the Discussion (in essence, the goal is to avoid eye-catching details that cannot be validated against the data), this initial concept of function *g*(*x*) is modified as follows. In a first step, function *g*_2_(*x*) is replaced by a function that is constant between *x* = 0 and the maximum at *x* = *θ* (indicated by the dashed line in Figure 2a). Renormalization (to get a probability density function again) yields:
3

In the next step, the distribution is discretized, taking into account that the MTR is an integer. Cases with an MTR not greater than 1 dB are finally combined with those showing no effect, and the resulting no-effect group is distributed equally over the MTR values - 1, 0, and 1 dB (Figure 2b). The last step has no other purpose than to facilitate the visualization of the model parameter *p*_0_ (which otherwise would be represented by a rather high peak).

### Modeling investigator bias

A deviation of the observed error distribution from a normal distribution will be interpreted as possible evidence of a partially biased practice on the part of the investigator. To corroborate the hypothesis, some modifications are applied to the above model. For a start, we confine ourselves to considering the threshold estimation for a single frequency. To mimic the common practice in clinical audiometry, the real-valued measurement error (normally distributed) is rounded to the nearest integer divisible by 5. Bias is introduced by assuming that an investigator sometimes reuses a previously estimated threshold instead of taking the time to carefully measure a small threshold change. To mimic this behavior in the model, a threshold difference of 5 dB between previous and current audiogram is ignored with a certain probability. Correspondingly, the model provides for the possibility that an investigator occasionally determines a threshold difference of 5 dB when a more careful procedure would have resulted in a threshold difference of 10 dB. It should be emphasized that the investigator is assumed to be unprejudiced as to the sign of the threshold change.

To simulate the estimation of MTRs, it was assumed that threshold estimations at different times (and possibly for different frequencies) have statistically independent measurement errors with identical standard deviations, *σ*. The difference between two threshold estimations for the same frequency (test-retest reliability), then, has the standard deviation 2^1/2^*σ*, and averaging 5 such differences (as required for obtaining the MTR) yields a measure with the standard deviation (2/5)^1/2^*σ*. The test-retest reliability of auditory threshold estimations has been investigated in many studies [25–29], and unlike in our model, the measurement error was found to be frequency-dependent. But this does not seriously compromise the validity of the model, because *σ*^2^ can be understood as the *mean* variance for the frequencies considered.

### Numerical calculations

All calculations were done with custom scripts using Matlab Version 7.14 (The MathWorks, Inc., Natick, MA, USA). The model parameters were optimized by least-squares fitting using the function FMINSEARCH (considering the cumulative distribution functions). ROC curves were calculated using the function PERFCURVE, which readily provides also the area under the curve (AUC).

The Monte Carlo simulations for the ROC analysis were done as follows. First, “true” MTR values were assigned to each of 100,000 cases so that the resulting cumulative distribution function was in accordance with that of the assumed model. Adding normally distributed random numbers to these values then yielded the “experimentally observed” MTR values.

## Results

### Measurement error

**Figure 3:**
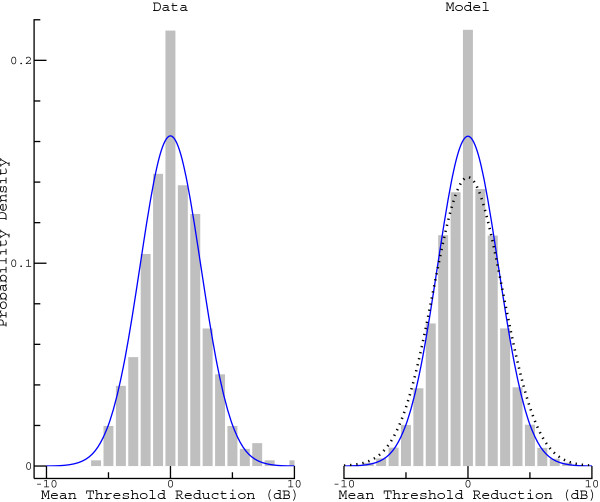
**Analysis of the measurement error.** The histogram on the left shows the MTR distribution estimated from the data, whereas the histogram on the right is based on a Monte Carlo simulation in which the investigator was assumed to be partially biased. The superimposed solid curves show normal distributions with zero mean and standard deviations corresponding to those calculated from the (measured or simulated) data. The normal distribution shown as a dotted curve in the right panel refers to the measurement error in the unbiased model.

Before attempts can be made to correct the distribution of observed threshold reductions for the measurement error, the latter has to be characterized. Our previous investigation (see Figure Four in [20]) suggested that the effect of glycerol barely intensifies after the third hour. Thus, the MTR distribution derived from the audiograms taken three and four hours after the glycerol intake (histogram on the left of Figure 3) is basically a fingerprint of the measurement error. Mean and standard deviation were calculated to be 0.26 dB and 2.45 dB, respectively (the curve superimposed on the histogram shows a normal distribution with a standard deviation corresponding to the calculated one, but with mean zero). The estimated mean confirms the previous observation that the threshold after 4 hours is only marginally lower than after 3 hours. The difference reached statistical significance, though (two-sided t-test yielded *P* = 0.045).

A remarkable feature of the estimated distribution is the pronounced peak at an MTR of zero, which is not fully compatible with the idea of a normally distributed measurement error. Although the reasons could be manifold, a Monte Carlo simulation using the model described in the Methods corroborates the hypothesis that this peculiarity reflects a methodological shortcoming: Knowledge of a previous audiogram biases the decision-making on part of the investigator. To obtain the histogram on the right of Figure 3, 100,000 partially biased investigations were simulated. A comparison with the histogram on the left shows that, by carefully adjusting the parameters, an excellent agreement between model and data could be achieved: It was assumed that single threshold estimations have a standard deviation of *σ* = 4.43 dB, that a threshold difference of 5 dB between previous and current audiogram is ignored in 80% of the cases, and that a threshold difference of 10 dB is reduced to 5 dB in 30% of the cases. Again, the solid curve represents a zero-mean normal distribution with a standard deviation corresponding to that estimated from the data (the simulated ones in this case). The dotted curve, by contrast, represents the distribution that, according to the model, would be obtained in the case of an unbiased estimation (as described in the Methods section, the standard deviation assumed for single threshold estimations, *σ*, was converted into the standard deviation of the MTR, yielding 2.80 dB). A comparison between dotted curve and histogram illustrates that greater threshold changes are slightly underrepresented in the latter.

### Frequency distribution of the mean threshold reduction

**Figure 4:**
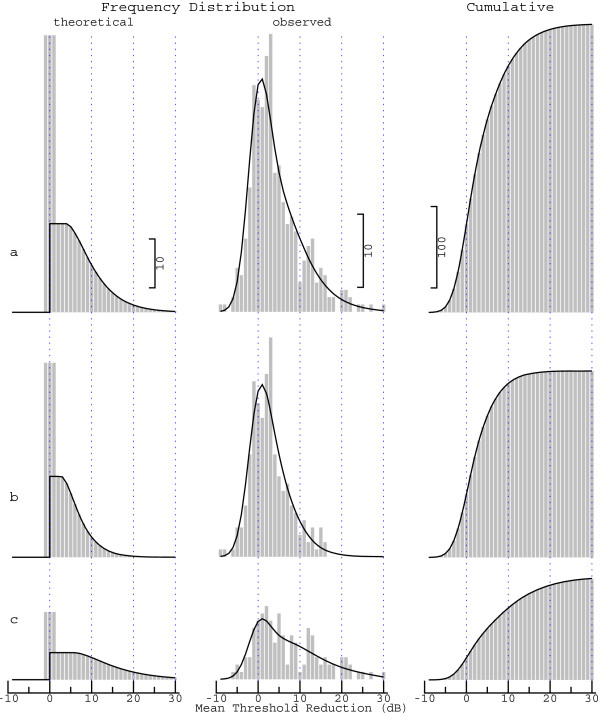
**Comparison between data and model for three groups of patients: (a) all patients, (b) the poor candidates for the glycerol test, and (c) the good candidates.** Convolving the theoretical frequency distribution shown on the left with the probability density function of the measurement error yields the observed frequency distribution shown in the middle (histogram representing the MTR values obtained from the data, curve representing the model). The corresponding cumulative frequency distributions are shown on the right.

For clinical testing, the audiogram obtained 4 hours after the glycerol intake is compared with the pre-test audiogram rather than the audiogram obtained after three hours (as in the error analysis above). The frequency distribution of the MTR calculated from these two audiograms is shown in the middle of Figure 4 (histogram). The three rows represent different groups of patients. In the upper row (a), all patients are considered, whereas the other two rows represent subsets of patients who either do (c) or do not (b) fulfill the rule of thumb proposed in our previous article [20]. According to this rule, the probability of a positive outcome of the glycerol test is increased if the mean low-frequency hearing loss in the pretest audiogram is within the range 30 to 70 dB and not smaller than the mean high-frequency hearing loss. Patients for whom the rule is satisfied will be referred to as the good candidates; the others will be referred to as the poor candidates, for the sake of convenience. Consistent with this idea, large MTR values (>15 dB) are found only for the good candidates (row c). But apart from that, the interpretation of the estimated distributions is complicated by the substantial blurring caused by the measurement error.

The goal of modeling is to eliminate the influence of the measurement error, i.e., to recover the distribution that would be obtained if hearing thresholds were determined with arbitrary accuracy. The result, represented by the histograms in the left column of Figure 4, will be referred to as the frequency distribution of the *true* MTR (the model parameters are provided in Table 1; the curves represent the function defined in Eq. (3)). A convolution of the theoretical distributions with the probability density function of the measurement error (curve on the left of Figure 3) yields the curves in the middle column, which agree reasonably well with the histograms derived from the data. If cumulative frequency distributions (right column) are considered instead of frequency distributions, the agreement between model and data appears to be almost perfect.

**Figure 5:**
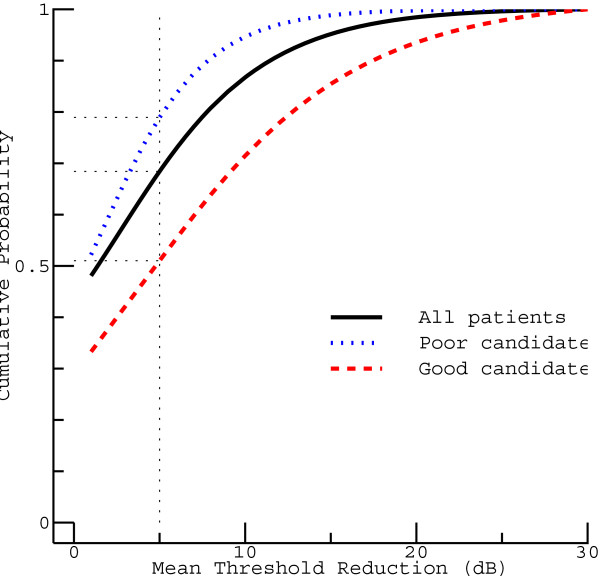
**Cumulative distribution functions for the true MTR.** These functions were derived from the cumulative distribution functions on the right of Figure 4 (which would be obtained in the absence of measurement errors). The solid curve represents all patients, whereas the other two curves represent the poor (dotted) and the good candidates (dashed).

Comparing the three groups of patients is facilitated when the differences in the number of cases are eliminated by normalization. The cumulative distribution functions in Figure 5 (obtained by rescaling the corresponding functions on the right of Figure 4) give the probability that the true MTR (which would be observed in the absence of measurement errors) does not exceed a specified value. If all patients are considered (solid curve), no or almost no effect (MTR ≤1 dB) is found in nearly every other case, while this applies to only every third of the good candidates (dashed curve). For the latter group, the cumulative distribution function increases relatively slowly, which contrasts with the steeper increase obtained for the poor candidates (dotted curve). As a consequence of these differences, the probability of finding an MTR of at most 5 dB (dotted vertical line) considerably varies for the three groups.

**Table 1:** **Model parameters and area under the ROC curve**

		Model parameters		Area under the ROC curve	
	N	*p*_0_	*θ* (dB)	assuming *σ* = 2.45 dB	assuming *σ* = 1.2 dB
All patients	354	0.378	3.89	0.922	0.976
Poor candidates	229	0.377	2.71	0.889	0.963
Good candidates	125	0.244	5.67	0.949	0.983

Three groups of patients are considered (N is the number of group members). In the middle, the values of the two model parameters, *p*_0_ and *θ*, are provided. On the right, the area under the ROC curve is given for two assumptions about the standard deviation of the measurement error.

### ROC curves

The performance of a diagnostic test is commonly characterized in terms of its specificity and sensitivity. If alternative versions of a method (or different methods) are to be compared, these performance measures are conveniently visualized in the so-called ROC space, where the horizontal axis represents the false-positive rate (1 - specificity) and the vertical axis represents the true-positive rate (synonymous with sensitivity). The analysis evidently requires that the test results can be checked against the actual facts or the results of a superior method serving as the “gold standard”. But this turns out to be problematic in the context of Menière’s disease. A Monte Carlo simulation based on the above modeling results offers at least a partial workaround.

**Figure 6:**
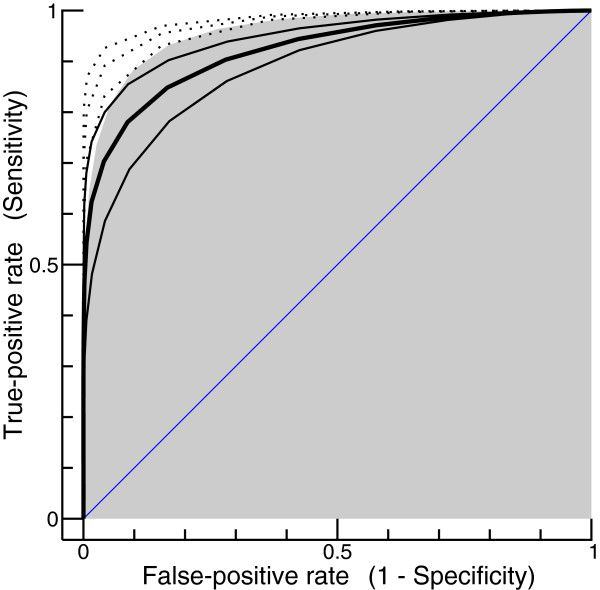
**False-positive rate (1 - specificity) versus true-positive rate (sensitivity).** The bold curve was obtained on the basis of all patients, whereas the curves above and below were obtained for the good and the poor candidates, respectively. Approximately halving the standard deviation of the measurement error yielded the dotted curves. By increasing the threshold of the assumed “gold standard” method from 2 dB to 5 dB, the bold curve turned into the curve bounding the gray area in the background.

To keep the simulation realistic, a “gold-standard” method is assumed to signal a positive glycerol effect if the true MTR exceeds a specified threshold (2 dB in our simulations, unless stated otherwise). The assumption of a threshold accounts for the fact that a distinction between “no effect” and “almost no effect” is not only difficult to accomplish in reality, but may also be irrelevant with respect to possible clinical consequences. After having defined a “gold standard”, a ROC curve [30–32] is easily derived from simulated data. The thick curve in Figure 6 was obtained using the model parameters that were determined on the basis of all patients, whereas the curves above and below were obtained using the parameters determined for the good and the poor candidates, respectively (see Table 1). The measurement error had a standard deviation of 2.45 dB, as estimated from our real data.

A convenient summary measure for the performance of a test is the area under the ROC curve (AUC). An intuitive interpretation of the AUC is as follows: If a randomly selected diseased individual is compared with a randomly selected non-diseased individual, the AUC corresponds to the probability that the test quantity (in our case the MTR) is higher for the diseased individual [33, 34]. Random guessing would result in a ROC curve corresponding to the diagonal line in Figure 6, which has an AUC of 0.5. By contrast, an AUC greater than 0.9 indicates a test of “rather high accuracy” [33]. The latter criterion is clearly fulfilled for the glycerol test, all the more if only the good candidates are considered (AUC values provided in Table 1). If methodological improvements allowed us to approximately halve the standard deviation of the measurement error (from 2.45 to 1.2 dB), the three dotted curves would be obtained instead of the three solid ones, and the AUC for the investigation of all patients would increase from 0.922 to 0.976.

The threshold of the “gold-standard” method in the above simulations (2 dB) corresponds to the lowest MTR value that, according to the model presented in Figure 2b, unequivocally represents a positive glycerol effect. But with respect to future applications it is conceivable that only patients showing stronger effects are considered good candidates for a certain clinical measure. This would require adjusting the criterion for a positive test result, which in our model is achieved by increasing the threshold of the “gold-standard” method. The curve bounding the gray area in the background of Figure 6 corresponds to the thick black curve (consideration of all patients), but the threshold was 5 dB rather than 2 dB. The differences between the two curves (the AUC increased from 0.922 to 0.960) have an obvious explanation: testing is the more accurate the greater is the effect to be detected.

## Discussion

### Modeling the glycerol test data

Central to this study was the attempt to explain our retrospective collection of glycerol test data [20] with a simple model that distinguishes between true effect and measurement error. The attempt turned out to be successful in that a model was found by which the cumulative frequency distribution of the observed MTR could be reproduced almost perfectly. Nevertheless, as subsequent considerations were based on the model rather than the data, a critical reflection on the model appears to be appropriate. The model builds on three main assumptions. *First*, the true MTR and the measurement error are assumed to be additive and statistically independent. Since the measurement error essentially reflects methodological imperfection and the patient’s uncertainty about the threshold, this point is not considered to be critical. *Second*, the measurement error is assumed to be normally distributed. Despite the minor problem revealed in Figure 3, this assumption is considered acceptable as well. A standard deviation of 2.45 dB for the mean of five threshold reductions suggests that the standard deviation of a single threshold reduction is 2.45 ⋅ 5^1/2^ = 5.48 dB. This value is consistent with the test-retest variability of audiometric thresholds reported by others [35–37]. *Third*, the probability density function of the true MTR is postulated to correspond to the template shown in Figure 2b. While the good agreement between model and data proves the suitability of this educated guess, a more meticulous examination is indispensable.

When trying to deduce the probability density function of the true MTR, it must be borne in mind that it is not about finding the unique solution to a well-posed problem. According to Eq. (1), the function sought, *g*(*x*), is convolved with the probability density function of the measurement error, *h*(*x*). The consequence is that finer details of *g*(*x*) are smoothed out, making a faithful reconstruction from the data impossible. This is why we chose a parameterized model. The law of parsimony, also known as Occam’s razor [38], mandates to make a model as simple as possible, and with only two adjustable parameters our model complies with this requirement. But still the problem remains that many different two-parameter models could explain the data equally well, for example the two models in Figure 2. A disadvantage of the first one (Figure 2a) is that the initial increase, from zero to the maximum, is an example of a fine structure that is inevitably smoothed out by the convolution with *h*(*x*). Moreover, the model suggests that patients without a glycerol-induced threshold reduction can be unequivocally distinguished from patients showing a rather small effect, which is, of course, unrealistic. As such aspects may lead to misunderstandings we switched to the model in Figure 2b. It is in the nature of the problem that there are alternatives to this second model, too. For example, one might consider smoothing the sharp transition that occurs around 2 dB. Questions of this kind become secondary, however, if the focus is on the *cumulative* distribution of the true MTR, because seemingly discrepant probability density functions may be associated with nearly identical cumulative distribution functions. Thus, given the fact that the model explains the data so well, the curves in Figure 5 can be assumed to provide a fairly realistic view of the cumulative distribution of the true MTR, even though details of the underlying probability density function are debatable.

### Performance of the glycerol test and future prospects

After having found a model that accurately reproduces the data, hitherto intractable questions could be addressed. In particular, defining a virtual “gold standard” allowed us to evaluate the performance of the glycerol test using ROC analysis. Even in its present form, the test turned out to have a “rather high accuracy” according to Swets’ [33] classification of diagnostic techniques. Reducing the standard deviation of the measurement error would further enhance the performance, although it is difficult to say how much improvement is realistically possible in a clinical setting. At least there can be no doubt that the current practice of determining thresholds of hearing in steps of 5 dB sets a lower limit for the size of effects that can be proven. Moreover, Figure 3 suggested that the investigator tends to be partially biased. Thus, innovative threshold estimation techniques such as the recently proposed single-interval adaptive procedure [39] could help to significantly amend the test.

It shall be emphasized that the performance measures examined in this study do not characterize the ability of the glycerol test to fulfill what Klockhoff [19] considered to be its genuine purpose: indicating endolymphatic hydrops. Instead, they refer to the capability of the audiometric procedure to detect a glycerol-induced threshold reduction. Admittedly, the original reason for configuring the analysis this way was a lack of reliable information about the presence or absence of hydrops, which necessitated finding a workaround. However, closer inspection suggests that our solution is not at all a substitute for a superior, albeit impracticable approach. This realization is linked to the key question as to what the actual purpose of the glycerol test is. Notwithstanding the above-mentioned later view, Klockhoff and Lindblom [40] took a positive glycerol test as evidence that hydrodynamic damping of the organ of Corti is reversible and that treatment with diuretic drugs may be of value. Treatment with diuretics is commonplace now, but strong evidence to support their use in Menière patients is limited [41]. Nevertheless, if not taken too literally, the initial idea of Klockhoff and Lindblom may also guide *future* clinical practice. What distinguishes the glycerol test from other approaches is that it does not simply measure the consequence of a pathophysiologic process, but probes to what extent the patient’s current medical condition responds to drug treatment, at least temporarily. Thus, the test could help to estimate the chances of success of pharmacological therapy [42, 43]. Progress as to that may, consequently, increase the interest in the glycerol test.

### Diagnostic testing for Menière’s disease from a more general perspective

Several other approaches have been proposed for diagnosing Menière’s disease. Probably the most popular technique at present is electrocochleography: Endolymphatic hydrops causes the summating potential (SP) to be enhanced compared to the compound action potential (AP) of the auditory nerve, yielding an increased SP/AP ratio [44]. However, opinions about the method are divided: A recent survey among American otologists and neurotologists showed that nearly half of the respondents had stopped ordering electrocochleography due to variability in results and lack of correlation with patients’ symptoms [45].

An abnormal endolymphatic pressure is supposed to affect also the impedance of the middle ear transmission system. However, testing for this effect by means of multifrequency tympanometry has only moderate diagnostic accuracy [46]. Another option for diagnostic testing seems to be the posture-induced phase shift of distortion-product otoacoustic emissions monitored around 1 kHz [47]. Auditory brainstem responses (ABR) have been studied as well. High-pass noise masking appears to be less efficient in patients with Menière’s disease [48]. Thus, these patients show ABR with abnormal latencies if the masking level is adjusted to suit normal hearing subjects [49]. The result of a traveling-wave-velocity test was reported to be correlated with the outcome of transtympanic electrocochleography [50].

The vestibular component of Menière’s disease can be tested by recording the vestibular evoked myogenic potential (VEMP), which, in the case of a unilateral manifestation of the disease, is of significantly lower amplitude on the affected side [51]. VEMP abnormalities may enable separation of Menière’s disease from other peripheral vestibulopathies [52, 53], although views differ as to whether Menière’s disease can be distinguished from vestibular migraine [54, 55].

This glimpse on recent studies shows that various possibilities are available to find objective correlates of Menière’s disease. Even though most of these techniques may not be suitable yet to provide reliable diagnostic information for individual patients, revealing statistical differences between groups of patients and working out the relationships between the different tests will help to better understand the disease.

Fukuoka et al. [56] recently compared MRI, electrocochleography, and the glycerol test in 20 patients diagnosed with definite Menière’s disease. While the latter two techniques yielded a positive result in only 11 and 12 patients, respectively, MRI gave evidence of hydrops in 19 patients. The authors therefore concluded that MRI is more useful for detecting hydrops than the two functional tests. Even taken together, the two functional tests were not competitive (only 15 patients showed a positive result in at least one test). This does not surprise considering that claims about the superiority of a combination of electrocochleography and glycerol test compared to the single tests [57, 58] are not well founded (false positives are left unconsidered).

Paradoxically, the seeming inferiority of the functional tests could eventually prove to be an opportunity. Diagnostic testing is most useful when the presence of disease is neither very likely nor very unlikely [59], and from this point of view, MRI is less informative than the functional tests: If finding endolymphatic hydrops in a patient diagnosed with definite Menière’s is rather likely, actually testing for the hydrops is wasteful unless there are compelling arguments to do so. Matters may be different if hydrops is considered in a more nuanced way, but attempts to derive a clinical benefit from this perception failed as yet: MRI neither predicted the outcome of intratympanic treatment with gentamicin [60, 61] nor demonstrated a reduction of hydrops after treatment with betahistine [62]. While it is questionable at this point whether any other presently available method would have been more successful in this respect, the examples illustrate that there are clinically important questions which imaging techniques may not be able to answer: A natural limit is reached when functional aspects without an obvious morphological correlate are concerned.

Although the upsurge of imaging methodology could eventually revolutionize the study of Menière’s disease, the above consideration shows that there is no reason to lose interest in functional methods. On the contrary, increased efforts should be made to improve them. As for the glycerol-induced change of state, it might be worthwhile to consider not only the threshold of hearing (classical glycerol test), but also other test quantities. And indeed, this idea has already been pursued regarding otoacoustic emissions [63], electrocochleography [64], and VEMP [65]. The ability to make useful predictions with respect to clinically important questions will ultimately decide which method (or what combination of methods) prevails. As to electrocochleography, it has been suggested, for example, that a high SP/AP ratio at the patient’s initial visit may be used as a predictor of poor hearing outcomes [66]. Admittedly, even more useful would be predictions about the chances of therapies being considered. But, at present, that would perhaps be asking too much, given that management of Menière’s disease is a topic which itself requires more research.

## Conclusions

The three key questions for decisions about using a diagnostic test are how accurate the test is, how it adds to the information provided by the history, examination and other (cheaper or more readily available) tests, and how it improves patient outcomes [13]. With regard to the various approaches that have been proposed for diagnosing Menière’s disease, these questions do not have simple, uncontroversial answers. Since different methods may target aspects of the disease that are not straightforwardly linked, premature conclusions about the relative merits of the various methods are to be avoided. This implies that defining a particular method as the “gold standard” is problematic unless the goal of diagnostic testing is clearly specified and the elected method is understood well enough to assess its suitability for that purpose.

While in the past the main focus was on getting indirect evidence of endolymphatic hydrops, MRI now provides a direct approach. However, if patients diagnosed with definite Menière’s disease almost always have endolymphatic hydrops, diagnostic testing with the goal to actually prove the hydrops may not be generally justified. Instead, more attention should probably be paid to the question as to what predictions can be made about the chances of specific therapies. The glycerol test (like similar tests using other diuretics such as furosemide [67] or urea [68, 69]) has the extraordinary property that it does not simply measure the consequence of a pathophysiologic condition in the inner ear, but investigates whether this condition is partially reversible. Even in its present, suboptimal form it fulfills Swets’ [33] criterion for tests of “rather high accuracy”. As a positive outcome proves the hearing loss to be partially reversible, the test could, prospectively, help to predict whether a patient is a suitable candidate for a certain type of therapy.

### Availability of supporting data

The data analyzed in this article are available from the Dryad Digital Repository (http://datadryad.org/resource/doi:10.5061/dryad.dr78n).

## Abbreviations

ABR: Auditory brainstem response
AP: Compound action potential
ATR: Aggregate threshold reduction
AUC: Area under the ROC curve
MRI: Magnetic resonance imaging
MRT: Mean threshold reduction
ROC: Receiver operating characteristic
SP: Summating potential
VEMP: Vestibular evoked myogenic potential.
